# Posttranslational Nitration of Tyrosine Residues Modulates Glutamate Transmission and Contributes to N-Methyl-D-aspartate-Mediated Thermal Hyperalgesia

**DOI:** 10.1155/2013/950947

**Published:** 2013-06-20

**Authors:** Carolina Muscoli, Concetta Dagostino, Sara Ilari, Filomena Lauro, Micaela Gliozzi, Erlisa Bardhi, Ernesto Palma, Vincenzo Mollace, Daniela Salvemini

**Affiliations:** ^1^Department of Health Sciences, University “Magna Graecia”, Edificio Bioscienze, Viale Europa, Campus Salvatore Venuta, Germaneto, 88100 Catanzaro, Italy; ^2^Interregional Research Center for Food Safety & Health (IRC FSH), Viale Europa, Campus Salvatore Venuta, Germaneto, 88100 Catanzaro, Italy; ^3^Drug Center, IRCCS San Raffaele Pisana, Via di Val Cannuta 247, 00163 Roma, Italy; ^4^University of Rome “La Sapienza”, Piazzale Aldo Moro 5, 00185 Roma, Italy; ^5^Department of Pharmacological and Physiological Science, Saint Louis University School of Medicine, 1402 South Grand Boulevard, St. Louis, MO 63104, USA

## Abstract

Activation of the N-methyl-D-aspartate receptor (NMDAR) is fundamental in the development of hyperalgesia. Overactivation of this receptor releases superoxide and nitric oxide that, in turn, forms peroxynitrite (PN). All of these events have been linked to neurotoxicity. The receptors and enzymes involved in the handling of glutamate pathway—specifically NMDARs, glutamate transporter, and glutamine synthase (GS)—have key tyrosine residues which are targets of the nitration process causing subsequent function modification. Our results demonstrate that the thermal hyperalgesia induced by intrathecal administration of NMDA is associated with spinal nitration of GluN1 and GluN2B receptor subunits, GS, that normally convert glutamate into nontoxic glutamine, and glutamate transporter GLT1. Intrathecal injection of PN decomposition catalyst FeTM-4-PyP^5+^ prevents nitration and overall inhibits NMDA-mediated thermal hyperalgesia. Our study supports the hypothesis that nitration of key proteins involved in the regulation of glutamate transmission is a crucial pathway used by PN to mediate the development and maintenance of NMDA-mediated thermal hyperalgesia. The broader implication of our findings reinforces the notion that free radicals may contribute to various forms of pain events and the importance of the development of new pharmacological tool that can modulate the glutamate transmission without blocking its actions directly.

## 1. Introduction

NMDARs in the spinal dorsal horns play a critical role in nociceptive transmission and modification [1, 2]. Glutamate-mediated activation of the NMDAR is fundamental in the development of hyperalgesic responses associated with pain of various etiologies [2–4]. Thus, the hyperalgesic responses detected in experimental models of acute inflammatory and neuropathic pain are blocked by intrathecal delivery of NMDAR antagonists [2, 3, 5–10].

We have reported that NMDAR activation releases superoxide (SO) which in turn is critical in mediating NMDA-mediated hyperalgesia [2, 11]. A key mechanism in maintaining and in sustaining high levels of SO at the sites of action is nitration of endogenous manganese superoxide dismutase (MnSOD), the enzyme that normally keeps SO under tight control [12]. Nitration and subsequent deactivation of MnSOD are carried out by PN [13–16], a product from the reaction of SO with nitric oxide (NO) [17]. NMDAR activation favors the accumulation of PN by forming SO [2, 11, 18–20] and NO simultaneously [21–23]. Moreover, Muscoli and coworkers demonstrated that SO-mediated nitration and deactivation of spinal MnSOD are a novel pathway of NMDA-mediated spinal hyperalgesia and hence of central sensitization since it helps to maintain high levels of SO that in turn maintains the nociceptive signaling [2, 11]. The goals of this study were to elucidate how elevated levels of SO maintain nociceptive signaling in response to NMDA. To this end, we focused on the potential role of nitration of key proteins involved in glutamate transmission, namely, NMDAR, glutamate transporter, and glutamine synthase (GS). cDNA cloning has revealed that the NMDAR is formed by several NMDAR subunits. The coexpression of GluN1 with various GluN2 subunits is required for a fully functional ion channel receptor and the combined expression of GluN1 with different GluN2 subunits results in a channel with distinct pharmacological and physiological properties that define NMDAR heterogeneity [24, 25]. PN interacts with the NMDAR leading to nitration of the tyrosine residues present on the NMDAR subunits. This is an irreversible reaction that leads to a constant potentiation of the synaptic currents and calcium influx and ultimately excitotoxicity [26–28]. It has been demonstrated that nitration of tyrosine residues in proteins is sufficient to enhance the degradation of the modified proteins by the proteasome *in vivo* [29] and could be a critical event also for the turnover of the receptors. Intrathecal administration of NMDA releases glutamate in the synaptic cleft [30–32]. Thus, thermal hyperalgesia, in response to intrathecal injection of NMDA, results from a persistent state of NMDAR activation due to high levels of glutamate in the synaptic cleft [3]. Once released, glutamate is not metabolized by extracellular enzymes but is removed by cellular uptake via glutamate transporters. GLT1, a selective glial cells transporter, possesses an intracellular domain rich in amino-acid residues susceptible to oxidation such as cysteines and tyrosines [33, 34]. PN nitrates the glutamate transporter lowering its capacity to remove glutamate from the synaptic space and leading to neurotoxic concentration of this neurotransmitter [2, 35–37]. Once glutamate is taken up into glial cells, it is converted into nontoxic glutamine by the glia-specific enzyme GS [38, 39]. Excitotoxic stimulation occurring in brain tissues seems to inactivate GS leading to reduced ability of astroglial cells to regulate glutamate turnover via GS activity [40–42]. Inhibition of GS activity increases central sensitization associated with inflammatory hyperalgesia, neuropathic pain, and opioid tolerance [37, 43–45].

The glutamate pathway proteins have key tyrosine residues which can be nitrated by PN: the net result of the posttranslational modifications of proteins involved in the tight regulation of glutamate homeostasis such as NMDAR, GLT-1, and GS provide a unifying link in signaling events underlying the central sensitization. Central sensitization is one form of long-term plasticity in the central nervous system. Sustained activation of primary sensory fibers supplying dorsal horn can induce long-lasting increases in the discharge amplitude of primary afferent synapses [46]. Central sensitization is an excitatory state of spinal cord dorsal horn neurons that transmit nociception due to increased responsiveness to suprathreshold and/or a lowered threshold to nociceptive signals; this manifests behaviorally as hypersensitivity to noxious (hyperalgesia) and nonnoxious (allodynia) stimuli. This state is a result of physiologic, biochemical, and molecular changes within spinal and supraspinal nociceptive modulating centers in the CNS and is partly responsible for chronic pain pathology [47].

The results of our studies demonstrate that NMDA-induced PN production maintains central sensitization and hyperalgesia by modulating glutamate transmission through posttranslational nitration of the NMDAR subunits, GLT1, and GS. 

## 2. Methods 

### 2.1. Animals

Male Sprague-Dawley rats (225–250 g, Charles River) used for these studies were purchased with intrathecally implanted cannulas (32 gauge, polyurethane). For the intrathecal catheters, briefly, the animal's head was flexed forward in the stereotaxic apparatus, an incision was made in the skin at the back of the head and neck, and the cisternal membrane was exposed by sharp dissection. The membrane was gently punctured with the tip of a #15 scalpel blade, and the distal end of a 7.5 cm long PE-10 catheter was passed through the opening in the cisternal membrane, into the intrathecal space. The catheter was loosely sutured to subcutaneous tissue, leaving the proximal end external to the animal and accessible to the experimenter, and the skin was then approximated using 4–0 absorbable sutures (Ethicon). All animals were housed and cared for in accordance with the guidelines of the University of Magna Graecia, Catanzaro, Italy, as well as complied with the Italian regulations for the protection of animals used for experimental and other scientific purposes (D.M. 116192), and with European Economic Community regulations. The rats were maintained in a controlled environment (12 h light/dark cycle, room temperature, 50–60% relative humidity). All experiments took place during the light period between 7:00 am and 10:00 am in a quiet room. 

### 2.2. Measurements of Thermal Hyperalgesia

Hyperalgesic responses to heat were determined as described by the Hargreaves method [48] and a cutoff latency of 20 sec was employed to prevent tissue damage in nonresponsive animals. Animals were allowed to acclimate for 30 minutes within a Plexiglas enclosure on a clear glass plate in a quiet testing room. A mobile unit consisting of a high intensity projector bulb was positioned to deliver a thermal stimulus directly to an individual hind paw from beneath the chamber. The withdrawal latency period of the right and left paw was determined to the nearest 0.1 sec with an electronic clock circuit and thermocouple. If the animal failed to respond within 20 sec, the test was terminated. Each point represents the change (sec) in withdrawal latency [(withdrawal latency of right plus withdrawal latency of left paw)/2] at each time point. Results are expressed as paw withdrawal latency (sec). After thermal testing, all the animals were sacrificed and the lumbar spinal cord (block from L4 to L6) was removed, immediately frozen in liquid nitrogen, and was randomly distributed for further analysis.

### 2.3. NMDA-Induced Hyperalgesia

Six groups were used.

*Group 1*. FeTM-4-PyP5+ Vehicle + NMDA Vehicle: animals (*n* = 8) received an intrathecal injection (10 *μ*L followed by a 10 *μ*L flush) of saline followed by an intrathecal injection of 10 *μ*L saline after 15 minutes which was followed by a 10 *μ*L flush of saline.

*Group 2*. FeTM-4-PyP5+ + NMDA Vehicle (FeTM-4-PyP5+ was tested at the highest dose, 2 nmol): animals (*n* = 8) received an intrathecal injection of FeTM-4-PyP5+ (2 nmol, 10 *μ*L followed by a 10 *μ*L flush) followed by an intrathecal injection of 10 *μ*L saline after 15 minutes which was followed by a 10 *μ*L flush of saline. 

*Group 3*. FeTM-4-PyP5+ Vehicle + NMDA: animals (*n* = 8) received an intrathecal injection (10 *μ*L followed by a 10 *μ*L flush) of saline followed by an intrathecal injection of NMDA (2 nmol in 10 *μ*L, [49]) after 15 minutes which was followed by a 10 *μ*L flush of saline.

*Groups 4–6*. FeTM-4-PyP5+ + NMDA (FeTM-4-PyP5+ was tested at 3 doses): animals (*n* = 8) received an intrathecal injection of 0.5, 1, and 2 nmol (10 *μ*L followed by a 10 *μ*L flush, *n* = 8 for each dose) of FeTM-4-PyP5+ followed by an intrathecal injection of NMDA (2 nmol in 10 *μ*L) after 15 minutes which was followed by a 10 *μ*L flush of saline.

 The thermal stimulus was applied separately to the right and left hind paw and paw withdrawal latencies were assessed immediately before and subsequently at 10, 20, and 40 minutes after NMDA injection. Results are expressed as Paw withdrawal latency (sec); a decrease in paw withdrawal latency relative to baseline is indicative of hyperalgesia. Determination of antinociception was assessed between 7:00 am and 10:00 am (light period). In the behavioural study, one person prepared the drugs and the other, blind to the drugs and dosage, ran the behavioural observation. The blind observer was identical throughout the study. 

### 2.4. Tissue Preparation for Cytosolic Extraction

For cytosolic extraction, tissues were homogenized with lysis buffer with a 1 : 3 w/v ratio. The lysis buffer (20 mM Tris-base, 150 mM NaCl, 10% glycerol, 0.1% Triton-X-100, 1% Chaps, 2 mM EGTA) contained 1% protease inhibitor cocktail (v/v). Solubilized extracts were sonicated (5 min) using a Sonicator (Fisher Scientific) and after 10 min of incubation in ice the lysates were centrifuged (12500 g, 30 min at 4°C). These supernatants were stored immediately at −80°C and were used to evaluate GS expression and activity. Protein concentration was determined using the Bicinchoninic Acid (BCA) protein assay (Pierce). All the experiments have been repeated at least twice for each different animal. 

### 2.5. Synaptosome Preparation

P2 membranes were obtained as described before [50]. Briefly, the lumbar tract of the spinal cord was homogenized in an ice-cold buffer (0.32 M sucrose, 100 *μ*M sodium orthovanadate, 0.02 M glycerophosphate, and 1% protease inhibitor cocktail, Sigma) in a glass homogenizer. The homogenates were centrifuged at 800 g for 10 min at 4°C. The resulting pellets were rehomogenized and centrifuged as before. The supernatants were combined and centrifuged at 12500 g at 4°C for 30 min to obtain the P2 pellet. This pellet was resuspended in homogenization buffer and protein concentrations were determined using BCA protein assay (Pierce). Samples were stored at −80°C and were used to determine NMDAR subunits and GLT1 expression following western blotting protocol as described below. All the experiments have been repeated at least twice for each different animal. 

### 2.6. Immunoprecipitation and Western Blot Analyses

Cytosolic fractions and P2 membranes obtained as previously described were used for immunoprecipitation and Western blot analyses. For immunoprecipitation 300 *μ*g of the solubilized proteins were incubated with 10 *μ*g of agarose-conjugated anti-nitrotyrosine antibody (Upstate Biotechnology) overnight at 4°C. Agarose beads were collected by centrifugation (1 min at 12000 ×g at 4°C) and washed in PBS (pH 7.4) three times. The mixture of the beads-antibody and binding proteins were resuspended in 50 *μ*L of sample buffer [2x, 0.5 M Tris*·*HCl, (pH 6.8) 2.5% glycerol/0.5% SDS/200 mM 2-mercaptoethanol/0.001% bromophenol blue] and heated at 95°C (5 min). To determine whether GS, GLT-1, and NMDAR subunits were nitrated, western blot of immunoprecipitated protein complex and total lysates were made using antibodies specific to these proteins. In brief, the samples were loaded in 10% SDS-PAGE minigels for GS detection and in 7.5% SDS-PAGE minigels for NMDAR and GLT1 detection (Bio-Rad). 

After separating by SDS/PAGE, proteins were transferred electrophoretically to nitrocellulose membranes (Bio-Rad). Ponceau red (Sigma) staining was used to ensure successful protein transfer. Membranes were blocked (1 hr, room temperature) with 1% Bovine Serum Albumin (BSA)/0.1% Thimerosal in 50 mM Tris*·*HCl, (pH 7.4)/150 mM NaCl/0.01% Tween 20 (TBS/T). Membranes were incubated with mouse monoclonal anti-GS (O/N, 4°C, 1 : 1000 dilution; Transduction Laboratories), mouse monoclonal GluN1 anti-body and rabbit polyclonal GluN2B (O/N, 4°C, 1 : 1000 dilution; Upstate Biotechnology), and rabbit polyclonal GLT1 (O/N, 4°C, 1 : 1000 dilution; US Biological). After washing with TBS/T, the membranes were incubated with anti-mouse horseradish peroxidase-conjugated secondary antibody or anti-rabbit horseradish peroxidase-conjugated secondary antibody (1 : 15000 dilution or 1 : 10000 resp.; Amersham) and the specific complex was detected by an enhanced chemiluminescence detection system (ECL, Amersham). Quantitation of nitration levels was then performed by densitometry using ImageQuant 5.2 software by Molecular Dynamics (Molecular Dynamics). Equal protein loading was determined using *β*-actin expression as housekeeping gene. SDS/PAGE was performed using 40 *μ*g of solubilized protein and subsequent transfer to nitrocellulose membrane (Bio-Rad). Membranes were blocked (1 h, room temperature) with blocking solution and then incubated with mouse monoclonal anti-*β* actin (2 h, room temperature, 1 : 5000 dilution; Sigma). After washing with TBS/T, the membranes were incubated with anti-mouse horseradish peroxidase-conjugated secondary antibody (1 : 15000 dilution; Amersham) and the specific complex was detected by an enhanced chemiluminescence detection system. No difference for *β*-actin was detected among the lanes. All the densitometry units have been normalized against actin for each lane and are expressed as the ratio of nitrated to unnitrated proteins.

### 2.7. Glutamine Synthase Activity

GS activity was determined using a Glutamine/Glutamate Determination Kit (Sigma) following the manufacturer's protocol. In brief, samples (25 *μ*L) in a final volume of 200 *μ*L were incubated with Acetate Buffer and Glutaminase for 1 hour at 37°C followed by incubation with Tris-EDTA-hydrazine buffer, NAD solution, ADP solution, and Glutamic Dehydrogenase for 40 minutes at room temperature. To evaluate the conversion of NAD^+^ to NADH an absorbance of 340 nm was imposed. All the experiments have been repeated at least twice for each different animal. 

### 2.8. Statistical Analysis

Results are given as mean ± SEM. Statistical analysis was performed using ANOVA followed by Student-Newman-Keuls. *P* < 0.05 was considered statistically significant.

## 3. Results

### 3.1. FeTM-4-PyP^5+^ Inhibits NMDA Mediated Thermal Hyperalgesia

**Figure 1:**
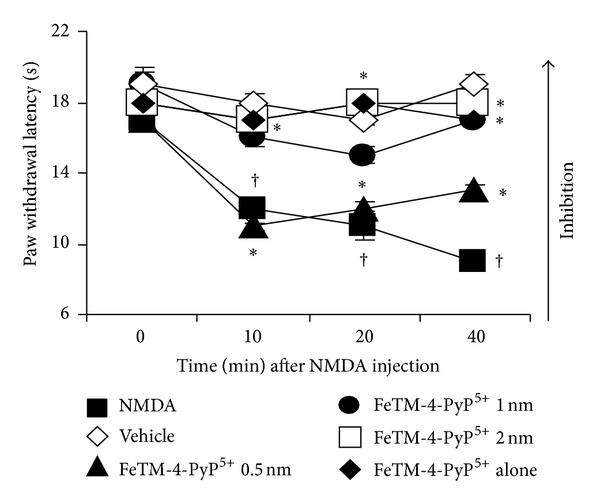
Intrathecal injection of NMDA (2 nmol, ■) causes thermal hyperalgesia when compared to vehicle *◊*, and this response is blocked by FeTM-4-PyP^5+^ in a dose-dependent manner (0.5 nmol (▲), 1 nmol (●), and 2 nmol (□), given intrathecally 15 min before NMDA). Intrathecal injection of FeTM-4-PyP^5+^ alone (*◆*, 2 nmol) did not exert any effect. Results are expressed as mean ± SEM for 8 rats; ^†^ *P* < 0.001 compared to vehicle and *P < 0.001 compared to NMDA alone.

Intrathecal injection of NMDA in rats (2 nmol; [49]) produces a time-dependent development of thermal hyperalgesia (Figure 1). Pretreatment of rats with the PN decomposition catalyst FeTM-4-PyP^5+^ (0.5–2 nmol, given intrathecally 15 minutes before NMDA) reduced the NMDA-evoked thermal hyperalgesia in a dose-dependent fashion (Figure 1). These results confirm our previous observations [11] and emphasize the fact that free radicals are important mediators of hyperalgesia induced by glutamate receptor activation. 

### 3.2. Intrathecal NMDA Induces Spinal GluN1 and GluN2B Tyrosine Nitration

**Figure 2:**
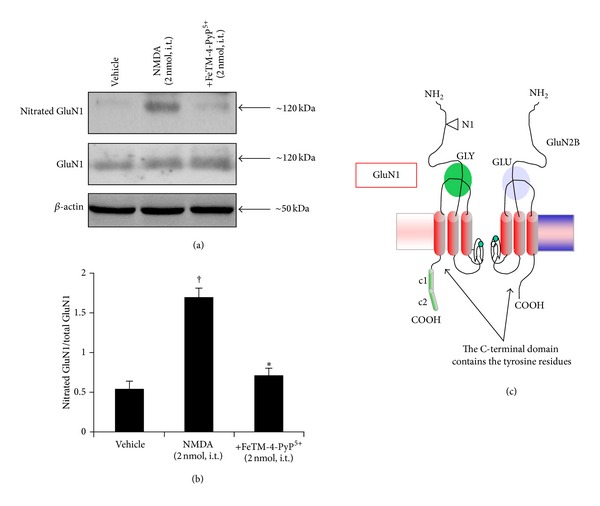
Inhibition of NMDA-induced hyperalgesia by FeTM-4-PyP^5+^ is associated with the inhibition of spinal protein nitration ((a)–(c)). As shown by immunoprecipitation, at the time of maximal NMDA mediated hyperalgesia (40 min), nitration of GluN1 was observed at the level of the spinal cord ((a), (b)). FeTM-4-PyP^5+^ (2 nmol given 15 min before NMDA) attenuates spinal GluN1 nitration ((a), (b)). Immunoprecipitation data shown in (a) are representative of at least 6 gels from 3 different animals performed on different days. Bar graph in (b) represents quantification by densitometric analysis. No difference for GluN1 or *β*-actin expression was detected among the lanes in these conditions. ^†^ *P* < 0.001 compared to vehicle and *P < 0.001 compared to NMDA alone.

**Figure 3:**
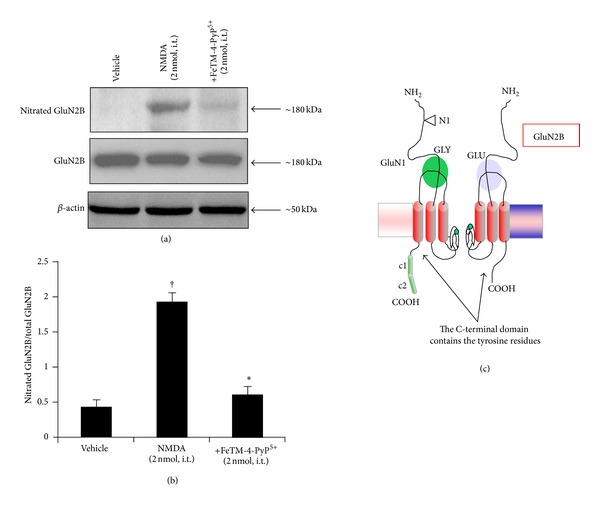
Inhibition of NMDA-induced hyperalgesia by FeTM-4-PyP^5+^ is associated with inhibition of spinal protein nitration ((a)–(c)). As shown by immunoprecipitation, the time at which the NMDA mediated hyperalgesia was at its peak (40 minutes), nitration of GluN2B was observed at the level of the spinal cord ((a), (b)). FeTM-4-PyP^5+^ (2 nmol given 15 min before NMDA) attenuates spinal GluN2B nitration ((a), (b)). Immunoprecipitation data shown in (a) are representative of at least 6 gels from 3 different animals performed on different days. Bar graph in (b) represents quantification by densitometric analysis. No difference for GluN2B or *β*-actin expression was detected among the lanes in these conditions. ^†^ *P* < 0.001 compared to vehicle and *P < 0.001 compared to NMDA alone.

Nitration of the tyrosine residues on the GluN1 (Table 1) and GluN2B (Table 1) subunits of the NMDAR occurred following thermal hyperalgesia that was induced by intrathecal injection of NMDA (2 nmol) as assessed by immunoprecipitation and western blot analysis (Figures 2 and 3). This effect was significantly reduced by pretreatment of the rats with FeTM-4-PyP^5+^ (2 nmol, given intrathecally 15 min before NMDA) (Table 1). 

### 3.3. Superoxide-Mediated Nitration of Glutamate Transporter GLT-1 Is Reversed by FeTM-4-PyP^5+^

**Figure 4:**
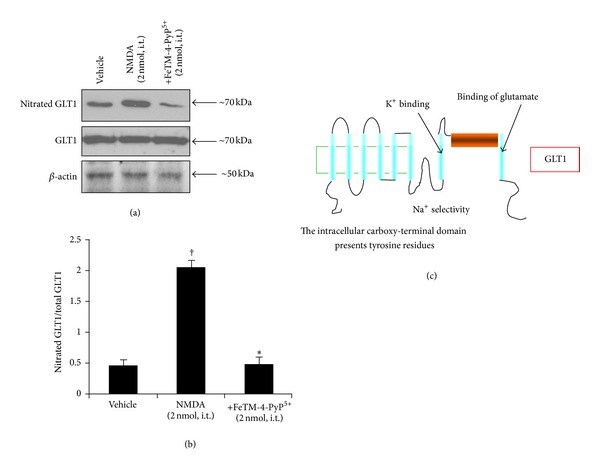
NMDA (2 nmol) induced thermal hyperalgesia was also associated with nitration of the glutamate transporter GLT1 ((a)–(c)). At the time at which hyperalgesia was at its peak (40 minutes), immunoprecipitation analysis revealed that FeTM-4-PyP^5+^ (2 nmol given 15 min before NMDA) reduced the NMDA mediated nitration of GLT1 at the level of the spinal cord ((a), (b)). Immunoprecipitation data are representative of at least 6 gels from 3 different animals performed on different days. Bar graph in (b) represents quantification by densitometric analysis. No difference for GLT-1 or *β*-actin expression was detected among the lanes in these conditions. ^†^ *P* < 0.001 compared to vehicle and *P < 0.001 compared to NMDA alone.

Intrathecal NMDA injection (2 nmol) leads to nitration of the glutamate transporter GLT1 observed by immunoprecipitation assay in the lumbar tract of the spinal cord (Table 1, Figure 4). Pretreatment of the rats with FeTM-4-PyP^5+^ (given intrathecally 15 min before NMDA) prevents GLT1 nitration (Table 1, Figure 4) together with the thermal hyperalgesia (Figure 1). 

### 3.4. Intrathecal NMDA Induces Nitration of Glutamine Synthase in Lumbar Tract of the Spinal Cord

**Figure 5:**
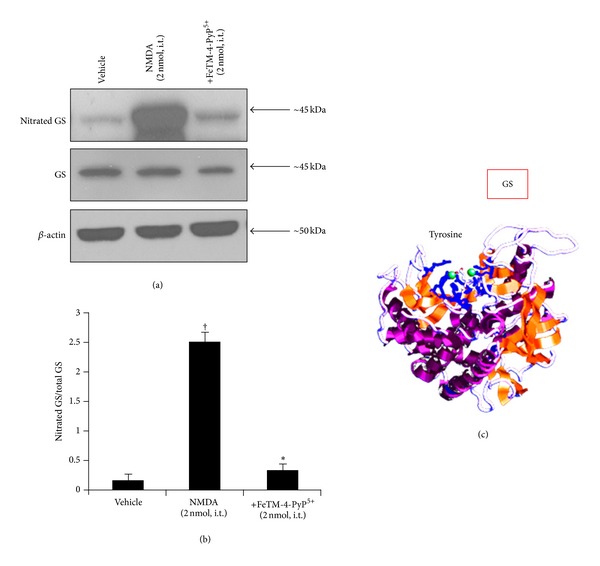
Nitration of glutamine synthase occurs following NMDA (2 nmol) induced thermal hyperalgesia ((a)–(c)). The time at which the NMDA mediated hyperalgesia was at its peak (40 minutes), immunoprecipitation analysis revealed that FeTM-4-PyP^5+^ (2 nmol given 15 min before NMDA) reduced the NMDA mediated nitration of GS at the level of the spinal cord ((a)–(b)). Immunoprecipitation data are representative of at least 6 gels from 3 different animals performed on different days. Bar graph in (b) represents quantification by densitometric analysis. No difference for GS or *β*-actin expression was detected among the lanes in these conditions. ^†^ *P* < 0.001 compared to vehicle and *P < 0.001 compared to NMDA alone.

**Figure 6:**
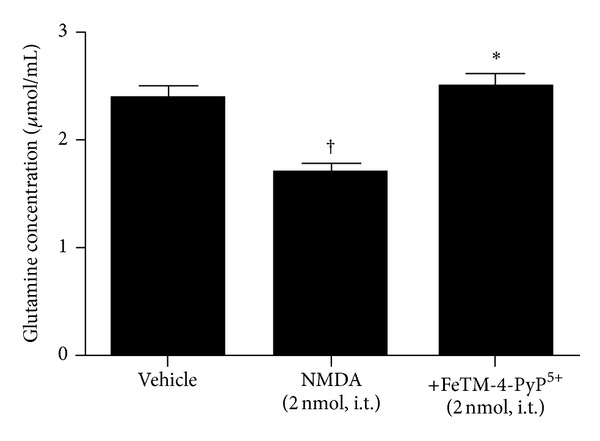
Inhibition of NMDA-induced hyperalgesia by FeTM-4-PyP^5+^ (2 nmol, given 15 min before NMDA) modulates GS activity. The amount of glutamine is highly decreased in animals treated with NMDA while it is restored by FeTM-4-PyP^5+^ treatment. Results are expressed as mean ± SEM for 3 rats; ^†^ *P* < 0.001 compared to vehicle; *P < 0.001 compared to NMDA alone.

In addition to NMDAR subunits and GLT1, the intrathecal NMDA injection (2 nmol) also induces nitration of the tyrosine residues of GS. This enzyme is found almost exclusively in astrocytes and normally converts the synaptically released glutamate into nontoxic glutamine. Tyrosine-nitrated proteins were immunoprecipitated and analyzed by western blot for the presence of nitrated GS. NMDA (2 nmol, given intrathecally) induces nitration of spinal GS (Table 1, Figure 5), and its inactivation was shown by a significant reduction of glutamine formation (Figure 6). FeTM-4-PyP^5+^ (2 nmol, given intrathecally 15 min before NMDA) blocked PN-mediated nitration (Table 1) and restored its enzymatic activity (Figure 6).

These data suggest that PN formation induced upon NMDAR activation leads to posttranslational modification of important proteins involved in the glutamate turnover contributing to the nociceptive pathway.

## 4. Discussion

The dorsal horn of the spinal cord is the site where the modulation of incoming pain information takes place through the release of glutamate by the C-fiber nociceptors. Here we have shown that once released, glutamate exerts its action on NMDAR increasing the production of reactive oxygen species such as SO, NO, and in turn PN which leads to nitration of tyrosine residues of key elements in the glutamate transmission. After intrathecal NMDA administration, nitration of NMDAR subunits, glutamate transporter GLT1, and GS synthase was observed in the spinal cord and these events were associated with enhanced hyperalgesic response to heat. 

The spinal cord neurons express three subtypes of glutamate receptors: the NMDA and the kainate (KA)/the AMPA (*α*-amino-3-hydroxy-5-methyl-4-isoxazolepropionic acid), which are both ligand-gated ion channels and the metabotropic receptors (mGluRs) [51]. On the other hand, the NMDAR activation is highly regulated and requires several conditions to occur. Channel opening needs the presence of depolarization, induced by the early activation of AMPA receptors by primary afferent fibres, in order to remove the magnesium physiological blockage that plugs the channel in a voltage-dependent manner [52] and the simultaneous activation by two agonists (glutamate and glycine) [51, 53]. The enhanced sensitivity of the postsynaptic cells evoked by glutamate, an event known as central sensitization, occurs by either the removal of the magnesium block in the NMDAR ion channel or via a posttranslational changes mediated by the phosphorylation of the receptor and this appears largely to be the mechanism involved in the maintenance of central sensitization [54]. The balance between phosphorylation/dephosphorylation on the tyrosine residues of the NMDAR subunits is known to regulate the activity of the receptor [55]. Receptor phosphorylation potentiates synaptic currents, calcium influx, and AMPA-receptor mediated responses known to be dependent on NMDAR activation [51]. Phosphorylation of the NMDAR on its tyrosine residues occurs via activation of Src kinase family, which is highly activated by PN [56]. Tyrosine nitration may keep the protein from performing the task of the phosphorylated form or it may mimic the structural changes imposed by phosphorylation and therefore imitate the consequences of phosphorylation with the difference that the nitration of the tyrosine residues is an irreversible process and can alter the normal protein's function by enhancing or inhibiting their activity [57]. NMDAR presents potential site for nitration and it has been previously demonstrated that the nitration of the NMDAR subunits observed *in vitro *and *in vivo *in a model of hypoxia leads to an increased glutamate binding to the NMDAR and consequently an increase in calcium influx and synaptic current [43, 58]. We have previously shown that during central sensitization there is an increased production of SO and in turn PN that leads to nitration and deactivation of MnSOD [2, 11, 59]. Deactivation of the endogenous scavenger of SO leads to an increased production of free radicals that, at least in part, contributes to the maintenance of the hyperalgesic state. 

Glutamate metabolism takes place only within the glial cells where the presence of specific transporters permits glutamate removal from the synaptic cleft. It is known that the uptake of glutamate by glutamate transporter system is impaired by PN [35, 37]. Most likely, loss in glutamate transporter activity is due to a posttranslational modification since it is neither associated with a decrease of mRNA nor to genomic mutations [60, 61]. PN lowers the capacity of the glutamate transporters to remove glutamate from the synaptic space leading to neurotoxic concentration of this neurotransmitter [35]. Dithiothreitol (DTT), a specific disulphide reducing agent and Mn(III) TBAP, a nonselective antioxidant, restored the transporter activity [62, 63]. Tyrosine nitration is a posttranslational modification that enhances susceptibility to degradation by the proteasome [29]. During NMDA-mediated hyperalgesia, we found that the transporter GLT-1 undergoes SO/PN attack that finally led to nitration of this protein.

Within the glial cells, glutamate catabolism occurs mainly via glutamine formation by GS which is the only enzyme in the CNS that is able to deactivate this excitatory amino acid. GS is inactivated by free radicals attack [64] leading to accumulation of synaptic glutamate and therefore prolonged NMDAR stimulation. Glutamate neurotransmission mediated via NMDAR plays a critical role in the development of central sensitization. Spinal release of glutamate and subsequent NMDAR activation favors PN accumulation by forming O_2_^∙−^ and NO simultaneously. Moreover, formation of NO, O_2_^∙−^, and PN in spinal cord contributes to the development of hyperalgesia that results from intrathecal delivery of NMDA [2]. GS activity is regulated by adenylation on the tyrosine residue in each of the 12 identical subunits of the enzyme [65]. Nitration of the tyrosine residues leads to complete loss of the catalytic activity of the adenylylated enzyme *in vitro *[65, 66] and loss of GS activity was observed during ischemia/reperfusion injury in a gerbil model [67]. During enhanced pain, neuroplastic changes occur in the spinal and supraspinal nociceptive modulating centers and may result in a hypersensitive state termed as central sensitization, which is thought to contribute to chronic pain states [47].

**Figure 7:**
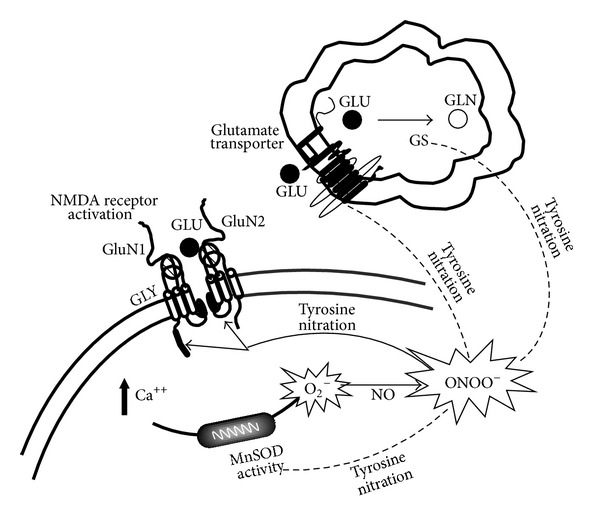
Nitration of tyrosine residues modulates glutamate transmission in the spinal cord. NMDAR activation increases intracellular calcium influx and leads to the production of peroxynitrite which in turn contributes to the hyperalgesic state by nitrating and subsequently activating NMDAR subunits while inhibiting GLT-1 and GS. Removal of PN by antioxidant abolished NMDA-mediated hyperalgesia by preventing tyrosine residues nitration of the glutamate pathway.

We have previously documented the role of PN in the nociceptive cascade [2, 11, 37, 44, 59, 68]. Here we have demonstrated that PN maintains central sensitization and hyperalgesia by modulating glutamate transmission through posttranslational nitration of the NMDAR subunits, GLT1, and GS. These events are fundamental for the regulation of glutamate turnover and consequently for the modulation of the spinal neurons responsiveness to the inputs that regulate the central sensitization as depicted in Figure 7. 

The broader implication of our findings is that PN may contribute to various forms of centrally induced hyperalgesia that are driven by NMDAR activation. This data together with our findings on the identification of free radicals scavengers as novel nonnarcotic agents [11, 37, 59, 69] strongly supports the notion that SO/PN is a viable therapeutic target for the development of nonnarcotic analgesics in pain of various etiologies. In fact, we observed that spinal administration of NMDA leads to GLT1, GS nitration and imbalance in glutamine production that is associated with development of thermal hyperalgesia. 

## Figures and Tables

**Table 1:** Densitometry data expressed in %.

	Treatment	Nitrated protein	Total lysate	*β*-actin
GluN1	Naive	17.00 ± 3.58	31.56 ± 7.8	32.26 ± 7.3
	NMDA (2 nmol, i.t.)	60.00 ± 2.94^†^	35.44 ± 5.5	33.53 ± 7.5
	NMDA + FeTMPyP (2 nmol, i.t.)	23.48 ± 1.43*	33.00 ± 3.5	34.21 ± 6.2

GluN2B	Naive	14.67 ± 1.96	34.00 ± 3.5	32.26 ± 7.3
	NMDA (2 nmol, i.t.)	65.58 ± 3.35^† ^	33.73 ± 3.7	33.53 ± 7.5
	NMDA + FeTMPyP (2 nmol, i.t.)	19.75 ± 2.28*	32.27 ± 2.5	34.21 ± 6.2

GLT-1	Naive	14.78 ± 2.34	34.22 ± 3.5	33.98 ± 2.3
	NMDA (2 nmol, i.t.)	70.31 ± 2.35^†^	34.36 ± 4.5	33.10 ± 5.8
	NMDA + FeTMPyP (2 nmol, i.t.)	14.91 ± 2.18*	31.42 ± 4.8	32.92 ± 6.8

GS	Naive	5.14 ± 1.12	33.10 ± 4.2	32.78 ± 4.4
	NMDA (2 nmol, i.t.)	84.46 ± 2.19^†^	33.40 ± 3.1	32.67 ± 6.7
	NMDA + FeTMPyP (2 nmol, i.t.)	10.40 ± 0.1*	33.50 ± 2.8	34.55 ± 4.3

^†^ *P* < 0.001 compared to vehicle and *P < 0.001 compared to NMDA alone.
